# Hemocytes released in seawater act as Trojan horses for spreading of bacterial infections in mussels

**DOI:** 10.1038/s41598-020-76677-z

**Published:** 2020-11-12

**Authors:** France Caza, Eve Bernet, Frédéric J. Veyrier, Stéphane Betoulle, Yves St-Pierre

**Affiliations:** 1 INRS-Centre Armand-Frappier Santé Biotechnologie, 531 Boul. Des Prairies, Laval, Québec H7V 1B7 Canada; 2 INRS-Centre Armand-Frappier Santé Biotechnologie, Bacterial Symbionts Evolution, Laval, Québec H7V 1B7 Canada; 3 Université Reims Champagne-Ardenne, UMR-I 02 SEBIO Stress Environnementaux et Biosurveillance des Milieux Aquatiques, Campus Moulin de la Housse, 51097 Reims, France

**Keywords:** Ecology, Marine biology, Innate immunity, Bacteriology

## Abstract

Global warming has been associated with increased episodes of mass mortality events in invertebrates, most notably in bivalves. Although the spread of pathogens is one of multiple factors that contribute to such mass mortality events, we don’t fully understand the pathophysiological consequences of sea warming on invertebrates. In this work, we show that in temperature stress conditions, circulating hemocytes in mussels leave the hemolymph to gain access to the intervalvar fluid before being released in seawater. External hemocytes can survive for several hours in seawater before entering other mussels. When infected by bacteria, externally-infected hemocytes can enter naive mussels and promote bacterial dissemination in the host. These results reveal the existence of a new opportunistic mechanism used by pathogens to disseminate in marine ecosystems. Such mechanisms may explain how thermal anomalies triggered by global warming can favor episodic mass mortality observed in recent years in marine ecosystem.

## Introduction

Because of their ability to accumulate xenobiotics in their tissues, their wide distribution, and their ecological importance, bivalve mollusks have long been recognized as good biological indicators for monitoring the effects of pollution and global warming in marine habitats^1^. They have also been exploited worldwide for their economic and nutritional values. Given their sensitivity to bacterial pathogens and development of different forms of cancer, including horizontally-transmitted leukemia^2,3^, there has been increasing interest in studying the physiology of their immune system, which has until recently received considerably less attention compared to other Metazoans.

All living organisms are constantly threatened by surrounding microorganisms seeking to exploit the same environmental niche. This is particularly true for invertebrates living in marine costal ecosystems where concentrations of bacteria are notoriously high. Survival and spread of marine bacteria, however, have to contend with several antagonistic factors including light exposure, nutrient deprivation, and physical and chemical properties of seawater^4^, notwithstanding viruses, which kill 20–40% of marine bacteria on a daily basis^5^. When entering into a host, bacteria further face the innate immune response, a crucial first line of host defense against pathogenic microorganisms. In bivalves, elimination of infectious pathogens by the innate immune system involves physical barriers, production of antimicrobial peptides, and phagocytes, which circulate freely in the hemolymph in direct contact with the animal's tissues where they perform local immune surveillance activities^6^. Phagocytic cells are also endowed with the ability to move to other compartments if necessary where they remain functionally competent, allowing them to perform a sentinel role similar to leukocytes in vertebrates^7,8^. Several studies have further shown that such migration of hemocytes through the pallial (or intervalvar) epithelium is bi-directional^9^. Unfortunately, for pathogens that resist phagocytosis or the action of hydrolytic immune mediators, such bi-directional movement provides an ideal gateway for establishing tissue-specific and/or systemic infection in the host^10^. This hypothesis is supported by recent studies showing that infection of hemocytes by some pathogens, such as Perkinsus marinus, can upregulate integrin-mediated cell motility of hemocytes, favoring their trans-epithelial migration and spread of pathogens inside the host^11^. Taken together, these studies show that movement of hemocytes in bivalves plays a central role in the infection process.

Mussel beds found in intertidal coastal shores are formed by dense aggregations of individuals held together by byssus threads. The density of mussel beds, which can reach up to thousands of individuals per square meter, varies considerably geographically and temporally depending of substrate type, slope and wave exposure^12^. Abundance of mussels, however, is very sensitive to abiotic stress factors that exert a significant impact on their community structure. Differences in resistance to thermal stress among species play a central role in controlling biodiversity within a given ecosystem^13^. This is particularly true among mussel populations which often cohabit in the same intertidal zones^14^. It is thus critical to better understand how environmental stress factors impact on the relationship between marine bacteria and invertebrates in marine coastal ecosystems and how such interplay may affect the community structure. Here, we report that hemocytes from mussels are released in seawater following temperature stress. Hemocytes can survive for several hours in seawater while remaining functionally competent before entering other mussels. We further show that opportunistic bacteria can infect hemocytes in seawater and take advantage of the entry of hemocytes into other mussels to spread into naive populations.

## Results

### Release of hemocytes in intervalvar fluids

**Figure 1:**
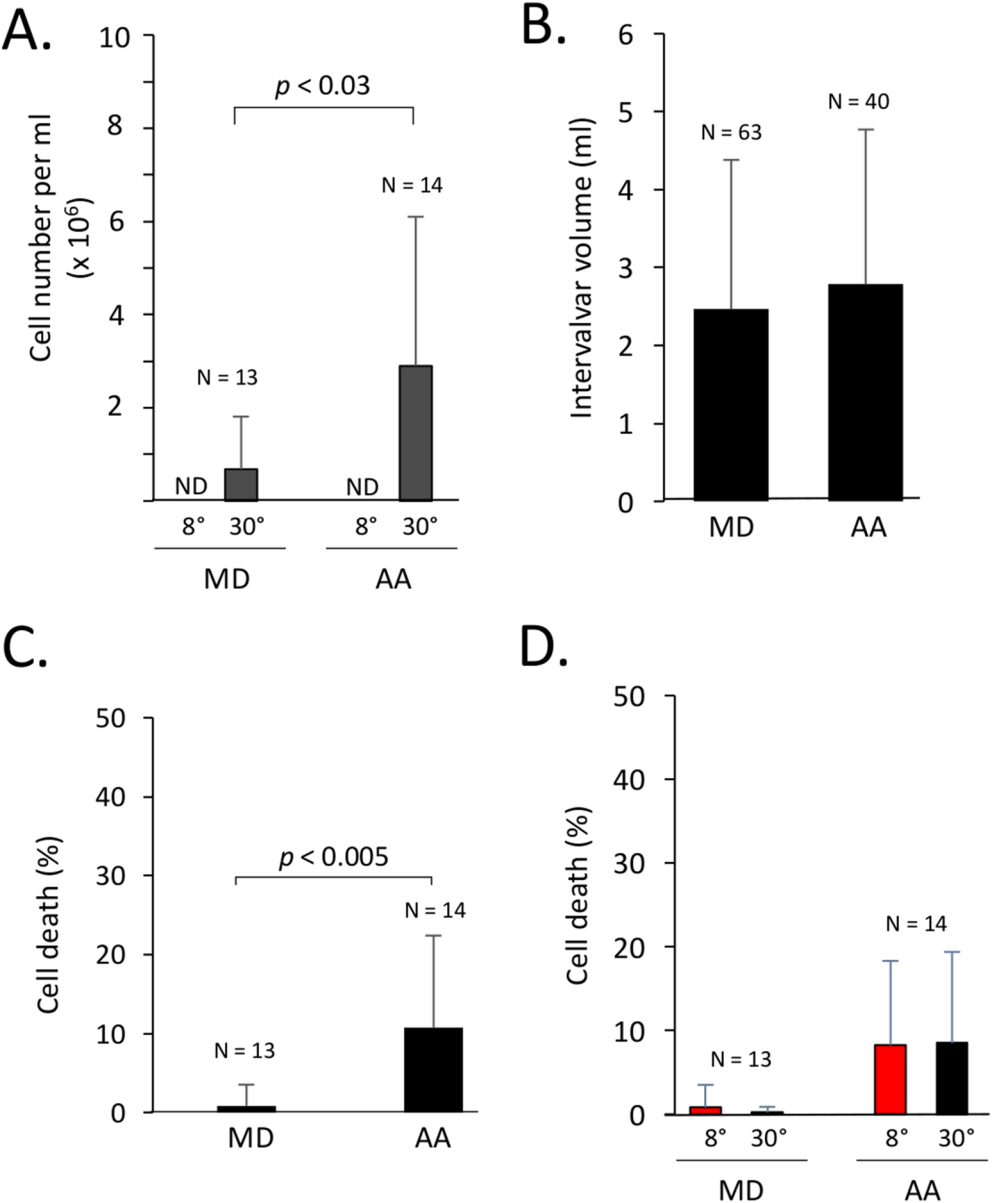
Release of hemocytes in the intervalvar fluid. (**A**) Number of viable hemocytes in the intervalvar fluid (IVF) of *M. desolationis* (MD) and *A. ater* (AA) immediately an acute temperature stress at 30 °C. (**B**) IVF volume collected from MD and AA immediately after the temperature stress. The results represent the pool of two independent experiments. (**C**) Viability of hemocytes found in IVF after the temperature stress as measured by trypan blue dye exclusion. The results represent the pool of five independent experiments. (**D**) Viability of hemolymphatic hemocytes collected immediately after the temperature stress (black bars). Controls were kept at 8 °C (red bars). Viability was measured by trypan blue dye exclusion test. *N.D.* non detectable.

This study was initiated during our research missions at Kerguelen Islands where we conducted experiments aimed at comparing temperature tolerance between two mussel species occupying the same habitat. During these experiments, we noticed that the intervalvar fluid (IVF), which normally contains very low numbers of hemocytes, was filled with hemocytes when mussels were immersed in 30 °C seawater for 30–90 min, an acute stress model used in other studies and this study to investigate the effect of temperature stress between mussels congeners^15–19^. This was particularly perceptible to the naked eye in the case of *Aulacomya ater (A. ater, *also commonly known as ribbed mussels or Magellan mussels) when the IVF was collected into clear polystyrene tubes. Further comparative analyses with *Mytilus edulis desolationis* (*M. desolationis,* also known as blue mussels) confirmed that viable hemocytes were present in IVF of both mussel species following an acute temperature stress (Fig. 1A). No significant number of hemocytes were detected in IVF of both *M. desolationis* and *A. ater* kept at 8 °C, the normal temperature of coastal marine ecosystems at Kerguelen. Concentrations of hemocytes in IVF of mussels incubated at 30 °C were significantly higher in *A. ater* compared to *M. desolationis*, independently of the IVF volume (Fig. 1B) and concentrations of hemocytes circulating in the hemolymphatic system (Supplementary Fig. S1). The viability (> 80%) of cells mobilized in IVF was comparable to that of hemolymphatic hemocytes (Fig. 1C,D). Analysis of the scattering profiles of hemocytes found in the hemolymph and the IVF were similar, suggesting that the passage of hemocytes from the hemolymph to IVF is not selective (Supplementary Fig. S2).

### Release of hemocytes in seawater

**Figure 2:**
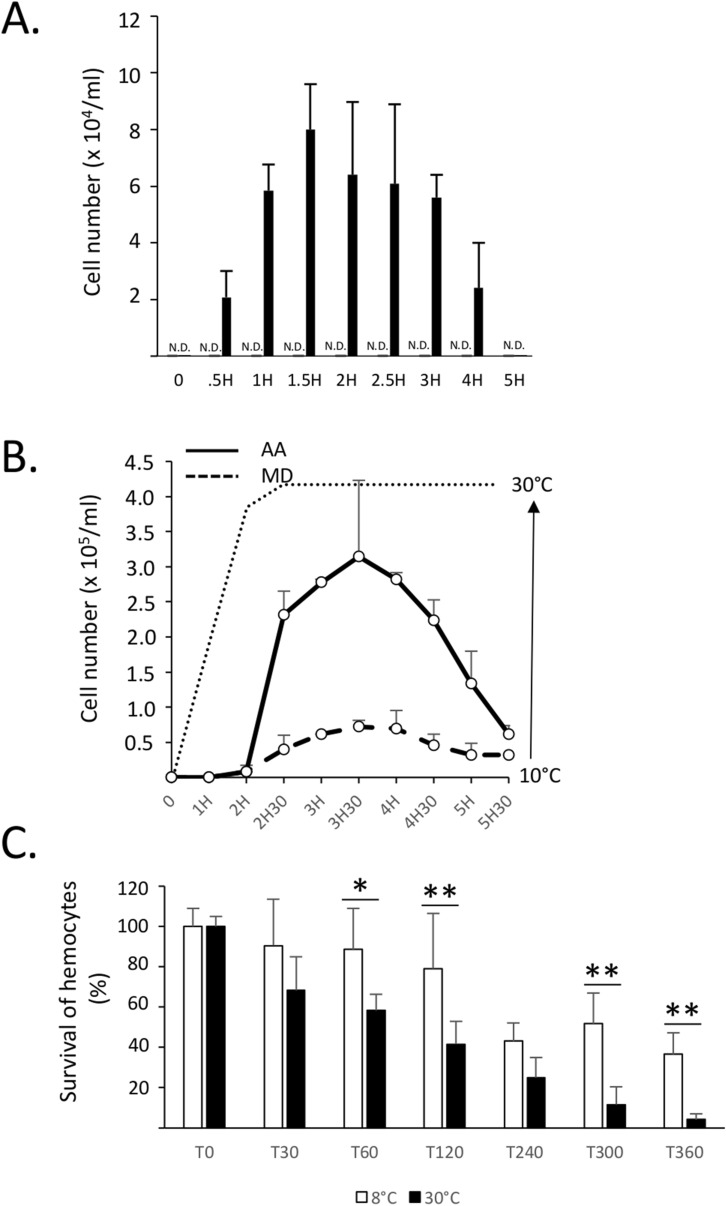
Release of hemocytes in seawater. (**A**) *A. ater* mussels (n = 6) were transferred in 1L tanks filled with seawater at 8 °C (grey bars) and 30 °C (black bars). At different times (minutes) post-transfer, aliquots (triplicates) of 0.5 ml of seawater were collected, centrifuged at low speed for 10 min at room temperature and resuspended in 50 μl of seawater before measuring cell counts in an hemacytometer by standard trypan blue exclusion method. These results are representative of three independent experiments. *N.D.* not detectable. (**B**) Comparative analysis of the number of viable hemocytes released in seawater by *M. desolationis* (MD) and *A. ater* (AA) (n = 10/group) after a progressive increase of temperature. The *x* axis indicates the times (hours) at which hemocytes were collected. The dotted line does represents the temperature (°C) at different times when hemocytes were collected. (**C**) Kinetics of survival of hemocytes of *A. ater* of in seawater at different times (min) after addition in tanks containing seawater at 8° and 30 °C. Each time point represents the mean of triplicates. Data are representative of two independent experiments.

**Figure 3:**
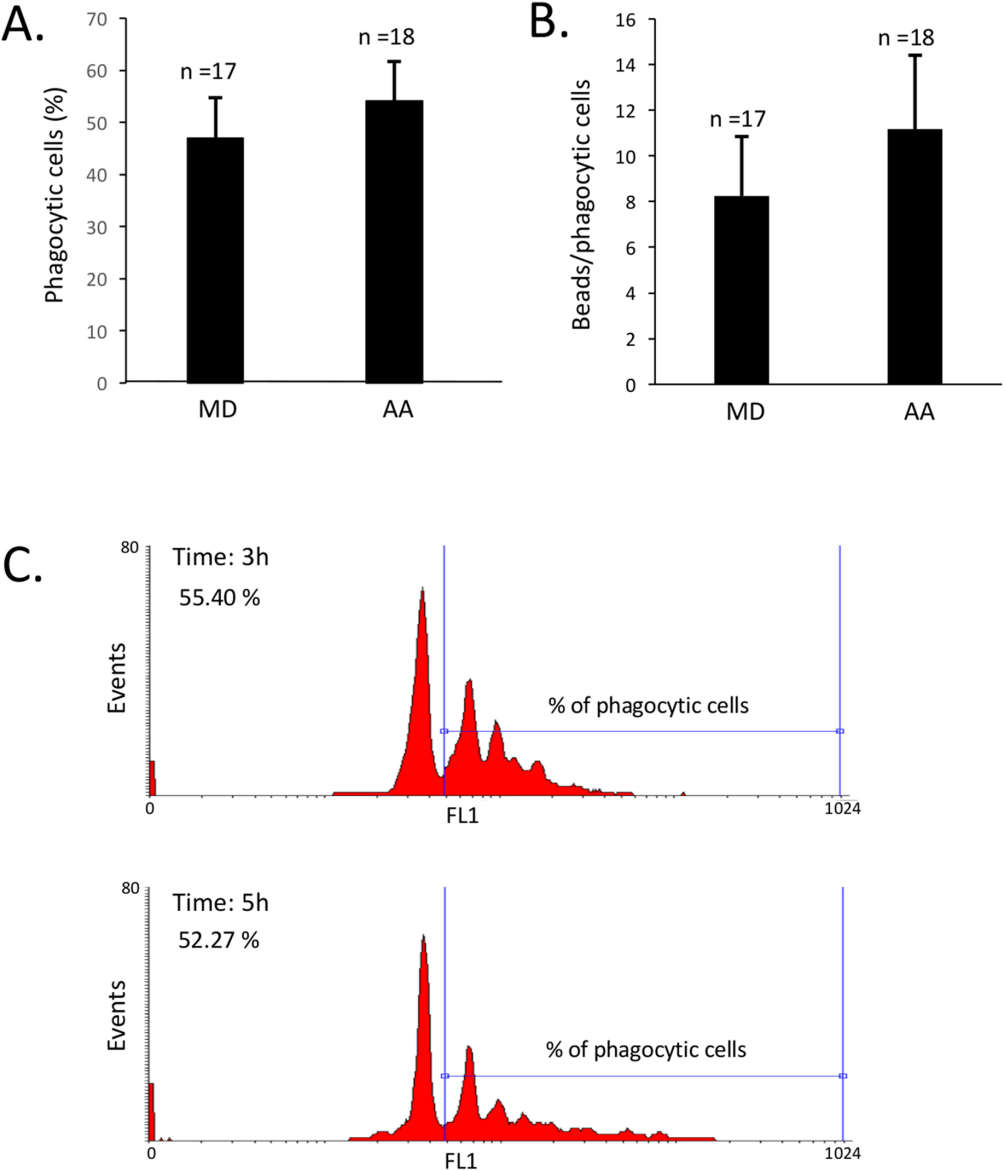
Phagocytic activity of hemocytes released in seawater. **(A)** Percentage of phagocytic cells and **(B)** number of beads phagocytosed by hemocytes released in seawater. The data represent the pool of measures (n = 17) taken between 0.5–6 h. The percentages of phagocytic cells and phagocytosed beads did not varied significantly within this interval of time for both mussel species. **(C)** Representative flow cytometric histograms showing phagocytic activity of hemocytes from *A. ater* released in seawater 3 and 5 h after thermal stress.

We next found that hemocytes located in IVF were subsequently released in seawater following the temperature stress (Fig. 2A). The number of hemocytes released in seawater was time-dependent and still detectable up to 4–5 h after the acute temperature stress. Viability of hemocytes released in seawater was confirmed by standard trypan blue exclusion method (Fig. 2A) and PI-staining (Supplementary Fig. S3). Release of viable hemocytes in seawater was also observed when the temperature was gradually increased to 30 °C (Fig. 2B). In the latter case, the release of hemocytes followed a similar pattern to that observed following an acute temperature stress, suggesting that hyperthermia rather than an acute thermal stress per se triggers the release of hemocytes. We also found that the release of hemocytes by *A. ater* was more pronounced when compared to *M. desolationis*, consistent with our previous observations showing higher hemocyte counts in IVF of *A. ater*. The survival of hemocytes in seawater was also time-dependent (Fig. 2C). Moreover, hemocytes in seawater were immune competent, at least in terms of phagocytic activity, and this for both mussel species (Fig. 3).

### Transfer of hemocytes into mussels

**Figure 4:**
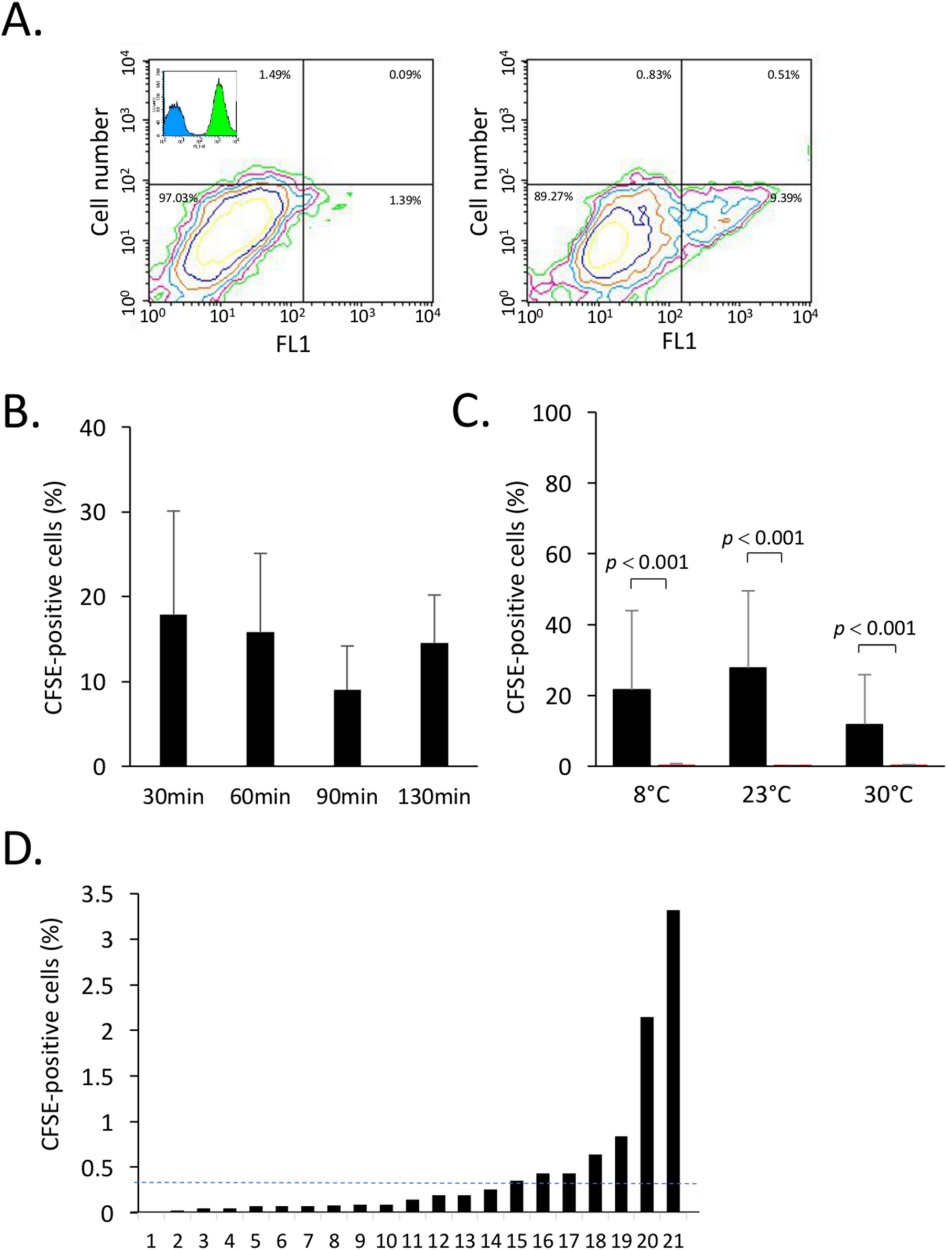
Entry of hemocytes in mussels. CFSE-stained hemocytes from *Mytilus spp.* were added to seawater. After the indicated times, IVF and hemolymph were collected and analyzed for the presence of CFSE-positive cells. (**A**) A representative contour plot showing hemocytes collected from IVF of mussels incubated without (*left*) and with CFSE-positive hemocytes (*right*). In the upper corner of the left contour plot, a representative overlay histogram of unstained (control) hemocytes in blue and CFSE stained hemocytes in green, (**B**) Effect of temperature on entry of CFSE-positive cells in IVF of mussels. (**C**) Percentage of CFSE-positive cells recovered in IVF of mussels at different temperatures 90 min after addition of CFSE-stained cells. The autofluorescence controls are shown in red. Its shows the autofluorescence as measured in (**A**) generated by unstained hemocytes recovered in IVF of mussels incubated in the same conditions. (**D**) Percentages of CFSE-positive cells present in hemolymph of *Mytilus *spp. maintained at 23 °C for 90 min after addition of CFSE-positive cells. The dotted line represents the average of CFSE-positive cells in hemolymph. The data are representative of two independent experiments.

Our observation that hemocytes are released in seawater and the fact that mussels have a natural tendency to open up their valve^20^ raised the possibility that hemocytes released in seawater may re-enter other mussels. To address this question, monodispersed suspensions of hemocytes were prepared from donor mussels and stained with CFSE, a dye that accumulates in the cytosol and which is commonly used for tracking cells in vivo by flow cytometry^21^ (Fig. 4A). CFSE-loaded hemocytes were then added to reservoirs containing mussels. At different times thereafter, IVF and hemolymph were collected from recipient mussels and analyzed by two-color flow cytometry for the presence of viable CFSE-positive hemocytes using the PI exclusion dye. Our results showed that CFSE-positive cells were readily detected in IVF of mussels 30 min after addition of CFSE-positive cells and did not vary significantly up to 90 min of incubation times (Fig. 4B). After 90 min, entry of CFSE-positive cells in IVF of recipient mussels was not significantly modulated by seawater temperature (Fig. 4C). Flow cytometric analysis of hemolymph collected from recipient mussels also contained CFSE-positive cells (Fig. 4D).

### Infection of mussels by *Mycobacterium marinum*

**Figure 5:**
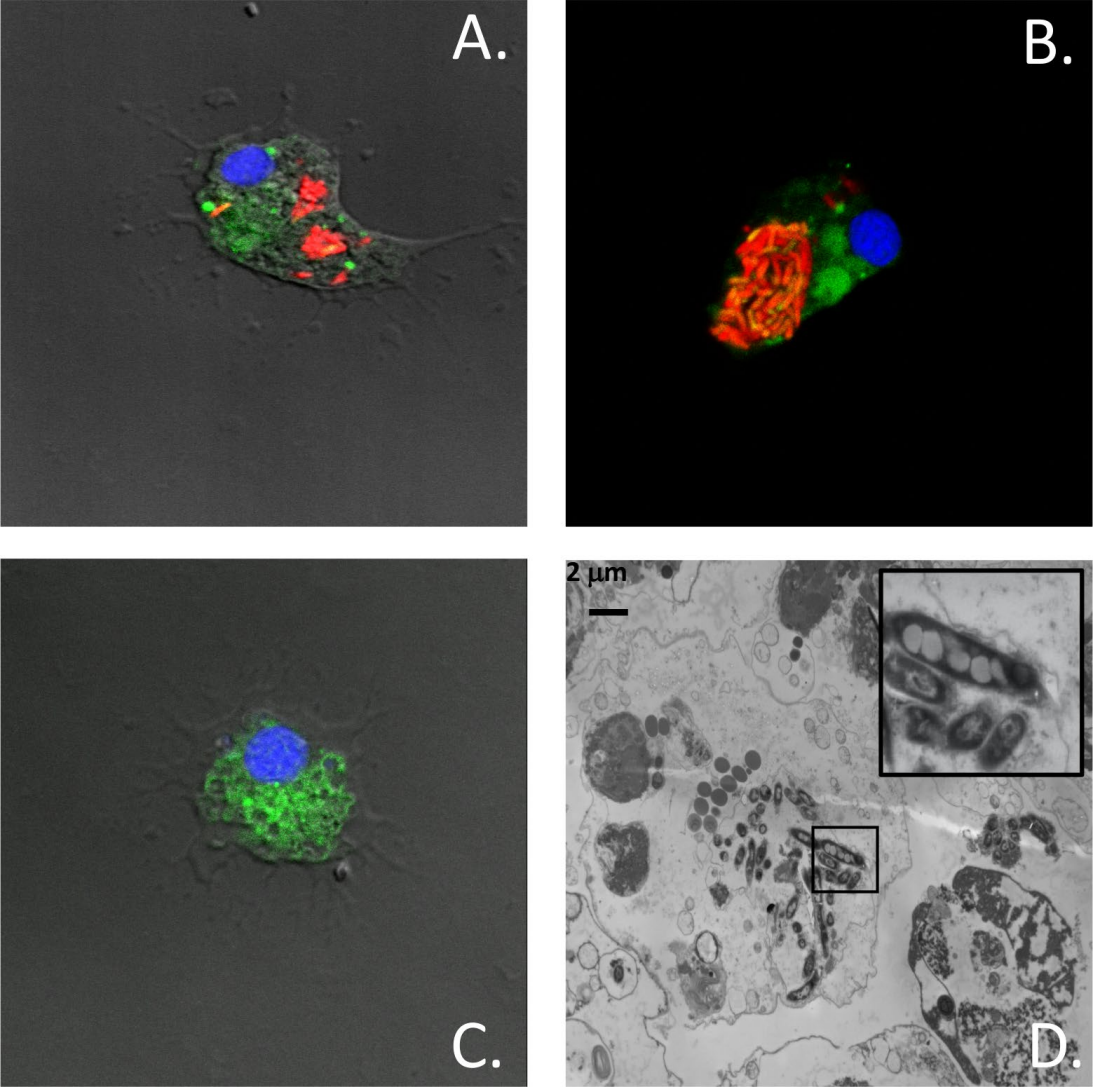
Infection of hemocytes by *M. marinum*. (**A**,**B**) Hemocytes from *Mytilus spp.* were infected with mCherry-expressing *M. marinum*. Cells were incubated with the CFSE and H33342 prior to analysis by confocal microscopy. **(C)** Uninfected hemocytes (Control). **(D)** Transmission electron microscopy showing a representative hemocyte infected with *M. marinum*. The insert on the upper right shows an enlarged area of the infected cell. The data are representative of two independent experiments.

**Figure 6:**
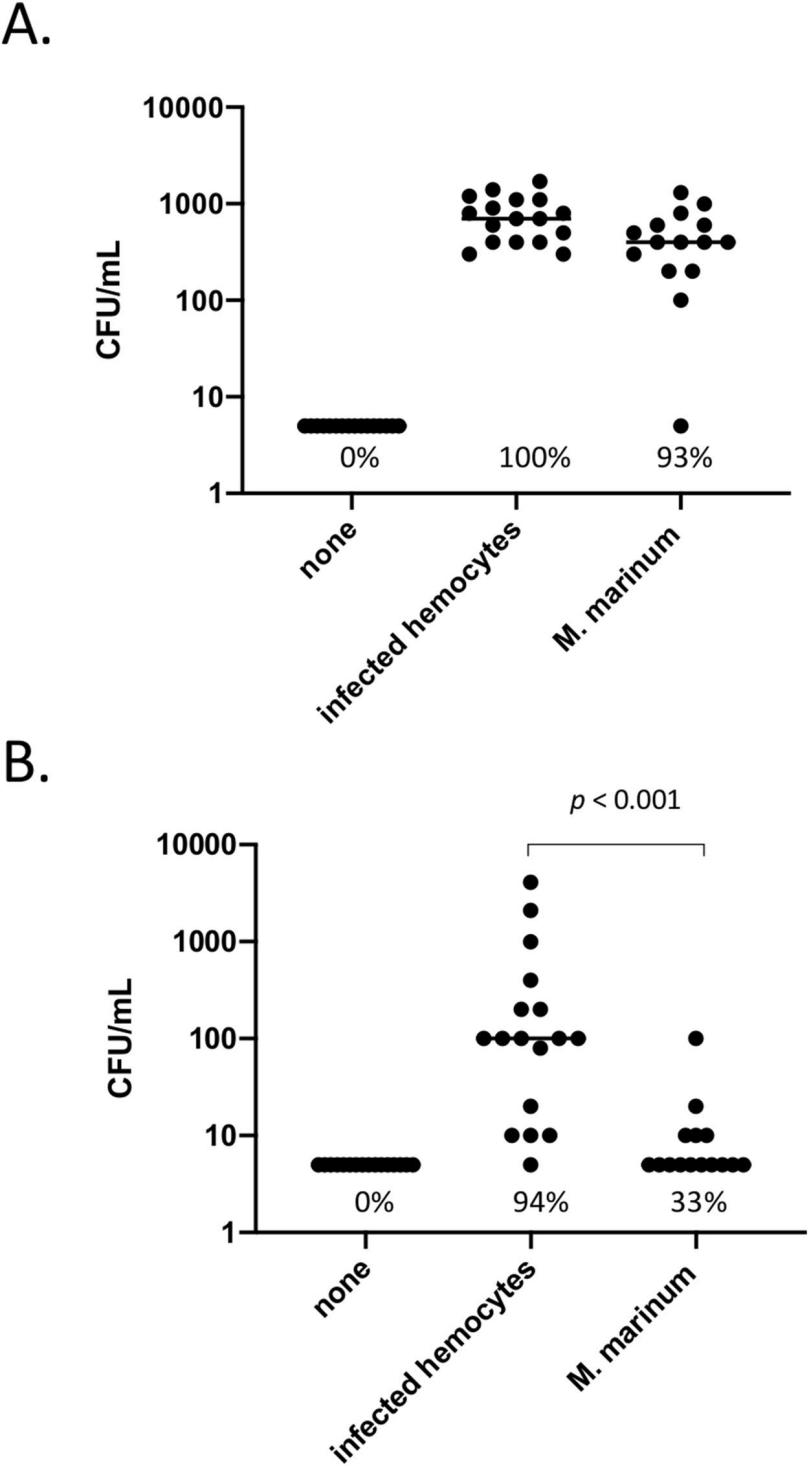
Hemocytes can serve as Trojan horses to infect mussels. Hemocytes infected by *M. marinum* or free bacteria were added to mussels. After 8 days, the number of bacteria in IVF **(A)** and hemolymph **(B)** were measured following CFU counts. Each point represents individual CFU counts. The data are representative of two independent experiments.

It is estimated that seawater contains more than 10^5^–10^6^ bacteria per ml^22,23^. Entry and spreading of bacteria in bivalves, however, faces a strong innate immunity following recognition of pathogens by immune cells and anti-microbial peptides. We thus investigated whether bacteria can take advantage of the release/re-entry mechanism to infect mussels. These studies were carried out upon our return from Kerguelen using *Mytilus *spp. As a model system, we used *Mycobacterium marinum*, a common bacterium found in bodies of fish and seawater^24^. This slow-growing bacterium is able to infect and survive for several days in macrophage-like cells including amoeba^25^. To facilitate tracking of *M. marinum-*infected hemocytes, a genetically engineered strain that constitutively expresses the mCherry fluorescent protein was used. We first established by confocal and electron microscopy that *M. marinum* can infect hemocytes (Fig. 5). A standard amikacin protection assay^26^ confirmed that *M. marinum* can enter, survive and multiply inside infected hemocytes (Supplementary Fig. S4). When infected hemocytes were added to reservoirs containing recipient mussels, we found that they were as effective as free bacteria to survive and grow in IVF (Fig. 6A). Hemolymph collected from recipient mussels further showed that infected hemocytes were significantly more efficient than free bacteria to establish a systemic infection in mussels (Fig. 6B). Taken together, these findings indicate that hemocytes released in seawater can serve as Trojan horses to infect mussels.

## Discussion

The idea that bacteria can use immune cells that circulate into the bloodstream to spread throughout the body, even in tissues that are protected by physical barriers, has been well documented. This is especially true for mycobacteria which infect and survive in phagocytic cells^27^. Here, our findings in mussels indicate the existence of a new opportunistic mechanism used by external bacteria to spread into naive hosts in marine ecosystems. This mechanism is initiated by a temperature- and time-dependent release of circulating hemocytes in IVF. Once in the IVF (also called “shell cavity”), hemocytes are released in seawater where they can survive for several hours while remaining functionally competent, at least in terms of phagocytic activity. This was observed for both *A. ater*, a mussel species only found in the Southern hemisphere, and in blue mussels (*Mytilus* spp.) present in both Southern and Northern hemispheres. We further showed that hemocytes in seawater can enter other mussels before gaining access to their hemolymphatic system. Finally, we found that hemocytes in seawater can be infected by *M. marinum* thereby providing an opportunity for the bacteria to enter and disseminate in naive mussels while being protected from the humoral and cellular components of the innate immune system. Taken together, these results suggest that temperature stress may play a critical role in spatial dynamics of pathogen propagation in marine ecosystems.

Although the circulatory system of bivalves is considered a semi-open system, intervalvar fluids of mussels normally contain very low numbers of hemocytes. Following a temperature stress, however, we found that hemocytes were released in IVF of both ribbed and blue mussels in Kerguelen Islands. Release of hemocytes in the IVF and outside the shell was particularly perceptible in *A. ater* compared to *M. desolationis*. Our findings are thus in line with previous studies showing that elevated temperature has significant effects on the physiology of mussels. Elevation of temperature, for example, increases the pumping rate of bivalves, including blue mussels^28^, possibly due to changes in physical/mechanical factors, such as water viscosity and/or oxygen depletion^29,30^. A previous study has shown that incubation of mussels at 33 °C induces behavioral responses, most notably rapid valve closure for prolonged periods^31^. Such thermal stress, while may not be as uncommon as we believe given that mussels, being dark in color, is rapidly warmed by the sun up to high temperatures during low tides. Considering the impact of climate change on marine coastal ecosystems, it is likely that exposure of intertidal mussels from Northern and Subantarctic seas to temperature of 30 °C and more will occur with increasing frequency. Several studies have also shown that such temperature stress induces molecular and cellular changes in hemolymphatic hemocytes^16,32^. It is thus logical to hypothesize that when the temperature reaches a critical threshold, hemocytes emigrate from the hemolymph to be located in IVF and, upon return to normal conditions or gape opening, they are released in seawater. Our experiments using *M. marinum* further support the idea that this mechanism could favor rapid propagation of pathogens in mussel beds. It was noteworthy that entry of bacteria in the hemolymphatic system was more efficient when the bacteria was inside the hemocytes compared to free bacteria. Future investigations will be needed, however, to determine how infected hemocytes trigger systemic infection in the host. It will be important to determine, for example, whether infected hemocytes cross epithelial lining to gain access to the vascular system and how and where the release of bacteria from infected hemocytes takes place. The results of this study, however, clearly show that temperature stress induces the release of hemocytes in seawater where they survive and are susceptible to bacterial infection, protecting bacteria which can now either enter the host via a Trojan horse mechanisms and/or in their free form. It is also possible that the passage of bacteria in hemocytes increases their virulence, as observed in marine marine microbes including several bivalve pathogens enhance their virulence in contact with host factors.This observation is in fact consistent with the ability of some bacteria to use phagocytic cells to evade the innate immunity. Such mechanism is particularly efficient in bivalves, and possibly other organisms without MHC class I proteins which normally trigger an adaptive immune response against non-self peptidic antigens. This could provide an explanation to previous studies suggesting that climate warming is associated with increased spreading of pathogens and episodic mass mortality in mussel beds^33–36^. The increase frequency of mass mortality events in marine coastal ecosystems is indeed attributed, at least in part, to increases of temperatures^37^. Our observation that release of hemocytes is more pronounced in *A. ater,* a cold-adapted mussel species*,* compared to *M. desolationis* further suggests that such response may impact local biodiversity of Subantarctic marine coastal ecosystems. Whether this explains the increasing dominance of blue mussels over ribbed mussels in specific regions of the Southern hemispheres remains an open question^14^.

Although we still ignore if the release of hemocytes in seawater and their re-entry into other individuals also occur in other bivalves, we do know that it occurs with *Mytilus *spp., suggesting that this migratory behavior of hemocytes is not restricted to mussel populations in Subantarctic regions but rather present in the Northern hemisphere as well. Future studies will be needed to determine how hemocytes proceed from the hemolymphatic circulation to IVF. Based on previous studies in humans and animals, it is logical to hypothesize that elevation of temperature may initiate a cascade of events that include adhesion of hemocytes to activated vascular cells before extravasation through a more permeable vascular system.

From a physiological perspective, our study raises the hypothesis that sharing of hemocytes among mussels may help build a “collective immunity”. On the one hand, entry of activated and functionally competent hemocytes secreting humoral mediators, such as soluble lectins, hydrolytic enzymes, pro-inflammatory factors^38^, specifically-oriented anti-microbial peptides^39^, may be used as an alarm signal to the colony to warn against environmental stress. Such sharing of "activated" hemocytes would serve to trigger the immune response more quickly in recipient mussels. In a way, it would be a horizontal transfer of a short term "immune memory". This possibility leads us to consider that mussel beds have their own immune repertoire and can develop some kind of a “herd immunity”. On the other hand, it may also be involved in the development of immune tolerance against specific bacteria, a form of immune memory of the innate response that has been highlighted following a secondary exposure of *Mytilus galloprovincialis* to *Vibrio splendidus*^40^. Such tolerance would favor systemic homeostasis with the goal of preserving energy, as suggested by studies in corrals^41^. Such sharing of hemocytes among mussels would not be, however, without a cost. Our study does indeed provide a mechanistic explanation underlying horizontal transmission of cancer of mussels in the Northern and Southern hemispheres, as recently reported^2,3,42^. Considering that transformation of cells significantly alters their morphology, it will also be interesting to determine whether cancer cells do behave differently compared to normal hemocytes. There are, however, multiple factors other than morphology that controls migration, including activation state, adhesion molecules, chemokine receptors, etc. Seuront et al.^43^ have observed that body temperature of Mediterranean blue mussels is frequently above 30 °C, and even more than 35–40 °C. It is logical to assume that global warming will favor external release of normal and leukemic hemocytes, thereby accelerating horizontal transmission of cancer in mussels.

In summary, our findings reveal the existence of a new mechanism triggered by elevated temperatures that may have significant impact on the dissemination of pathogens, and possibly other diseases, in marine coastal ecosystems. Such mechanisms could explain how thermal anomalies triggered by global warming may favor episodic mass mortality in mussel beds observed in recent years.

## Methods

### Mussels

Adult specimens (55–70 mm length) of *Mytilus edulis desolationis* (*M. desolationis*) and *Aulacomya ater* (*A. ater*) were collected on the intertidal rocky shore of Port-aux-Français (049°21.235S, 070°13.490E) at Kerguelen Islands in December 2018. Mussels were sampled in the intertidal zone and kept in a container containing seawater from the sampling site. The water was oxygenated by aeration and organisms were maintained throughout the transport phase at a water temperature closed to that measured on field (+ 7.5 °C). Mussels were immediately transferred to the marine laboratory of Port-aux-Français and placed in temperature-controlled (8 °C, pH 7.7, salinity ~ 34 ‰) aerated 50 l tanks containing filtered recirculating seawater maintained on a 12 h:12 h light/dark cycle. Other adult blue mussels (*Mytilus *spp., 55–70 mm length) were obtained from a commercial supplier (PEI Mussel King Inc., Prince Edward Island, Canada) and placed in a temperature-controlled (4 °C) aerated tank containing 20 l of 32‰ artificial saline water (Reef Crystal artificial marine salt, Instant Ocean, VA, USA). For each experiment, individuals shell lengths and weight were measured.

### Collection of hemocytes from intervalvar fluids and hemolymph

Unless otherwise indicated, hemocyte samples were collected from single individuals, without pooling and centrifugation. The intervalvar fluid (IVF) was first carefully and rapidly removed with the tip of a knife to minimize contamination with extrapallial fluids and collected into 15 ml sterile Falcon tubes. Hemolymph was withdrawn from the adductor muscle using a syringe fitted with a 25 gauge needle. The number of viable cells present in cell suspensions was determined by the standard trypan blue exclusion method and/or by flow cytometry using propidium iodide (PI).

### Phagocytosis

Phagocytosis assays were carried out as described^19^. Briefly, hemocyte suspensions (1–5 × 10^5^ cells) were distributed in round-bottomed 96-well plates. Yellow-green latex FluoSpheres (Molecular Probes Inc., Eugene, OR, USA; diameter 1.2 μm) were then added to each well at a hemocyte:bead ratio of 1:100. Unless otherwise indicated, all experiments were carried out at 20 °C in the dark. After 18 h, supernatants were removed by gentle flickering and the plates tapped dry on absorbent paper. Cell pellets were resuspended in 0.2 ml of filtered seawater and the percentage of cells containing fluorescent spheres was determined by flow cytometry using a FACSCalibur flow cytometer (Becton Dickinson, Mountain View, CA, USA) equipped with an air-cooled argon laser, providing an excitation at 488 nm. Hemocyte cell populations were defined based on their forward (FSC) and right angle (side) scatter (SSC) properties. A FSC threshold was used to eliminate cell debris and bacteria. Fluorescence emission was collected at 520 nm. The cytometer was usually operated at a medium flow rate. A total of 5 000–10 000 events was acquired for each sample and stored in the list mode data format. A phagocytic index was measured as the percentage of cells that had ingested fluorescent beads. The phagocytic capacity of hemocytes was also measured as the mean number of engulfed beads within the phagocytic hemocytes population as described^14^.

### Thermal stress and hemocyte transfer experiments

Following acclimatization, mussels (n = 6) of similar length were transferred into 1L tanks containing oxygenated seawater at 30 °C or, as controls, at 4 °C (*Mytilus *spp.) or 8 °C (*M. desolationis and A. ater*). To measure the release of hemocytes in seawater, at different times (minutes) post-transfer, aliquots of 0.5 ml (triplicates) of seawater were collected, centrifuged at 280×*g* for 10 min at room temperature and resuspended in 50 μl of seawater. This cell suspension was used for measuring the number of viable cells present in cell suspensions by by light microscopy using standard trypan blue exclusion method and to measure their phagocytic activity as described above.

### Tracking of hemocytes

Hemocytes were collected from 60 to 100 blue mussels (*Mytilus *spp.) and subsequently washed in seawater by low speed centrifugation at room temperature. To measure survival of hemocytes in seawater at different temperatures, monodispersed cell suspensions were obtained and cells added to 1L tanks (final cell concentration: 0.5 × 10^6^ cells/ml). At the indicated times, aliquots (0.5 ml) were collected, centrifuged at 280×*g* for 10 min at room temperature and resuspended in 50 μl of seawater. This cell suspension was used for measuring the number of viable cells present in cell suspensions by light microscopy using standard trypan blue exclusion method and for measuring phagocytic activity by flow cytometry, as described above. For measuring entry of hemocytes into mussels, hemocytes were stained with 5(6)-Carboxyfluorescein diacetate *N*-succinimidyl ester (CFSE, Molecular Probes, Invitrogen, Eugene, OR) at a final concentration of 10^6^ cells/ml following the manufacturer’s instructions. After an incubation of 10 min in the dark, cells were washed three times, resuspended and added to 1L tanks containing oxygenated seawater at the indicated temperatures obtain a final concentration of 0.5 × 10^6^ cells/ml. A total of 15–20 adult mussels (*Mytilus *spp.) were then added to each tank. At different times post-transfer, mussels (n = 5) were removed and their IVF and hemolymphatic cells collected, washed in seawater, and resuspended in seawater for analysis by flow cytometry as described above. A total of 1000–5000 events was acquired for each sample and stored in the list mode data format for analysis using the CellQuestPro software.

### Infection of mussels by *Mycobacterium marinum*

Mussels were acclimated for two days before the start of experiment. Hemocytes were collected from adult *Mytilus *spp. as described above, seeded in six-well plates (10^6^ cells/well) and incubated with Penicillin–Streptomycin (Pen-Strep) for 24 h to eliminate most of the bacteria, washed three times with seawater. After 24 h, hemocytes were infected with *M. marinum* expressing mCherry^44^ for 6 h at a multiplicity of infection (MOI) 10:1. A standard amikacin protection assay^21^ was performed by adding amikacin (200 µg/ml) during 1 h to kill extracellular bacteria. Subsequently, infected-hemocytes were washed three times with seawater and incubated for different time (0 h, 24 h, 48 h in Supplementary Fig. S4). Cells were lysed with one ml of Triton 0.1% (v/v) and detached using a sterile cell-scraper. Bacteria were counted using serial dilution on 7H10 agar plate containing hygromycin (50 μg/ml). Colonies on plate were counted after 2 weeks of growth.

### Confocal microscopy

Hemocytes infected with *M. marinum* genetically engineered to express mCherry constitutively were stained with CFSE and Hoescht 33342 (LifeTechnologies) and fixed with paraformaldehyde 2% (v/v). Fixed cells were mounted on microscope glass slides using ProLong Gold antifade reagent (Invitrogen). Images were acquired in sequential scanning mode on an LSM780 confocal microscope (Carl Zeiss Microimaging) every 370 nm, at a resolution of 200 nm, 63X microscope objective. The 3D images adjustments were as follow: zoom of 3,16 Pixel (0.04 mµ) and a pinhole of 1 airy.

### Electron microscopy

Cells were fixed in 2.5% glutaraldehyde in cacodylate buffer at pH 7.4 with 0.2 M sucrose overnight and then post-fixated in 1.33% osmium tetroxide in Collidine buffer pH 7.4 for 1 h at room temperature. After dehydration by successive passages through 25, 50, 75, 95% and 100% (twice) solutions of acetone in water (for 15–30 min each), samples were immersed for 16–18 h in Spurr:acetone (1:1 v/v). Samples were then embedded in BEEM capsules using Spurr’s resin (TedPella) before incubation at 60–65 °C for 20–30 h. After polymerization, samples were sliced using an ultramicrotome (LKB Brooma—2128 Ultratome) and were put onto Formvar and carbon coated-copper 200-mesh grids. Samples were contrasted with 5% uranyl acetate in 50% ethanol (v/v) for 15 min followed by lead citrate for five minutes. Cells were visualized using a Hitachi H-7100 transmission electron microscope with AMT XR-111 camera.

### Infection of mussels by *Mycobacterium marinum*

For horizontal transfer experiments, mussels were reared in 10 l polycarbonate tanks (30 mussels/tank) containing seawater with aeration at room temperature. The seawater was renewed daily. The algae *Platymonas helgolandica* var. *tsingtaoensis* and *Isochrysis zhanjiangensis* were supplied to the mussel as food source as previously described^45^. Mussels were acclimated for 1 week before the start of experiment. In parallel, hemocytes were isolated and infected with *M. marinum* as described above. Infected hemocytes were then added to a reservoir containing mussels. A second (control) group of mussels were challenged with *M. marinum* previously treated with amikacin for 24 h at room temperature. After 2 h, mussels were removed, washed in seawater and re-cultured for 8 days at room temperature. Another control group included mussels not infected with *M. marinum*. At 8-days post-challenge, hemolymph and intervalvar fluids were collected, centrifuged to collect cell pellets and processed for CFU counts as described above.

### Statistical analysis

Statistical significance of the experiments was evaluated using the unpaired Student’s t-test or the Fisher’s exact test. Results were considered statistically significant at P ≤ 0.05. The non-parametric Mann–Whitney U test was used for comparison of data that were not normally distributed. All data are expressed as the mean ± S.D. n represents the number of experiments on individual animals.

## Supplementary information

Supplementary Information.

## Abbreviations

*A. ater*: *Aulacomya ater*
*M. desolationis*: *Mytilus edulis desolationis*
*M. marinum*: *Mycobacterium marinum*
IVF: Intervalvar fluid
