# Supplementary material for: Hemocytes released in seawater act as Trojan horses for spreading of bacterial infections in mussels
Source: Sci Rep. 2020 Nov 12;10:19696. doi: 10.1038/s41598-020-76677-z (PMC7665017; doi:10.1038/s41598-020-76677-z)
Supplement: Supplementary file 1 — Supplementary Information. [file 41598_2020_76677_MOESM1_ESM.pdf]

Hemocytes released in seawater act as Trojan horses for spreading of bacterial infections in mussels.

France Caza[1], Ève Bernet[2], Frédéric J. Veyrier[2],
Stéphane Betoulle[3], and Yves St-Pierre[1]

[1]INRS-Centre Armand-Frappier Santé Biotechnologie, Laval, Québec, Canada, H7V 1B7.
[2]INRS-Centre Armand-Frappier Santé Biotechnologie, Bacterial Symbionts Evolution, Laval, Québec, Canada, H7V 1B7.
[3]Université Reims Champagne-Ardenne, INERIS, SEBIO UMR I 02, 51097, Reims, France

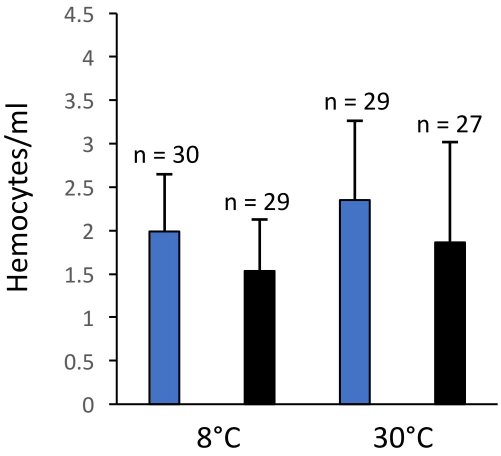

Supplementary figure 1: Hemolymphatic hemocytes counts in MD (blue) and AA (black) at 8° and immediately after a 30°C thermal stress. The results represent the pool of three independent experiments. No statistically signifcant differences were observed.

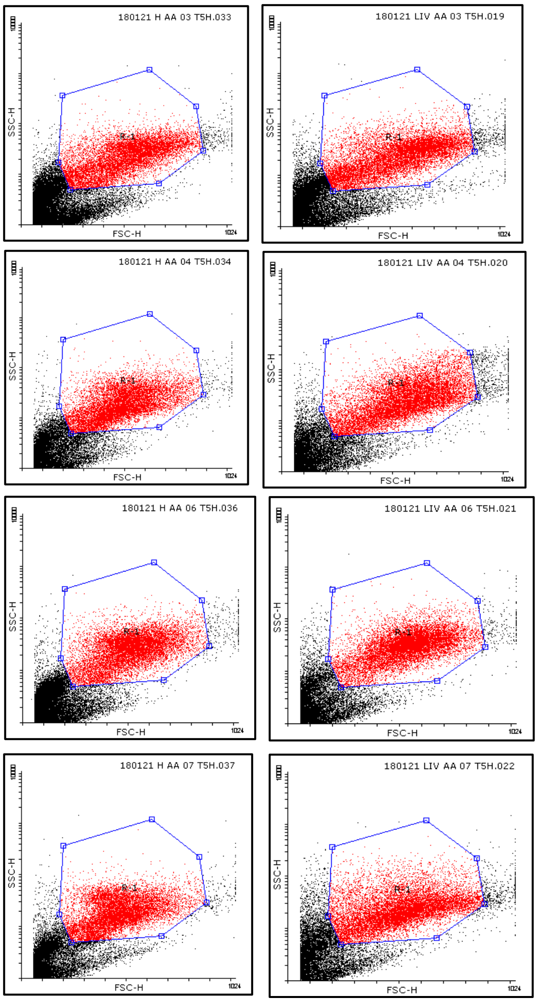

Supplementary figure 2: Scattering profiles of hemolymphatic hemocytes and hemocytes released IVF. The forward scatter (FCS) and side-scatter (SSC) histograms were obtained from four individual *A. ater* at 5h post-temperature stress

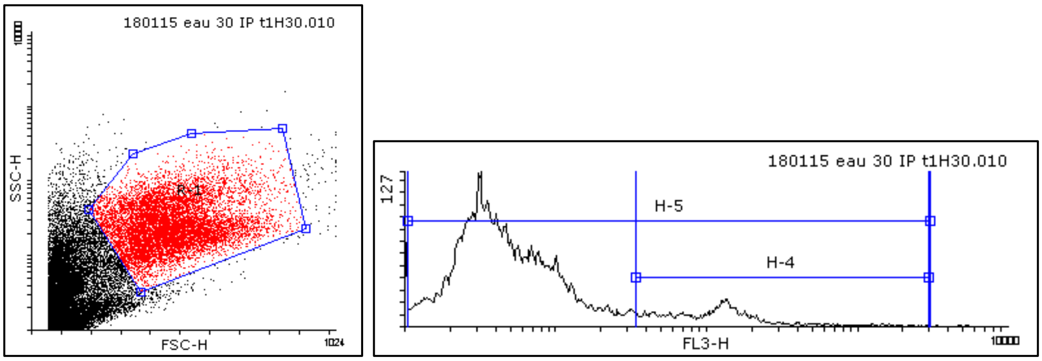

Supplementary figure 3: Viability of hemocytes released in seawater. Representative forward scatter (FCS) and side-scatter (SSC) histograms (*Left*) and fluorescence (FL3, *Right*) from PI-stained hemocytes collected in seawater at 90 min post-temperature stress of *A. ater*.

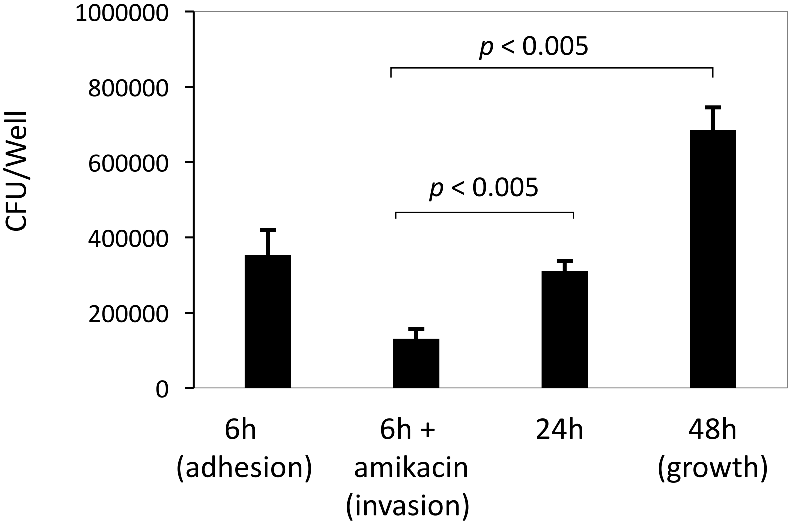

Supplementary figure 4: *M. marinum* can enter, survive and multiply inside hemocytes. Hemocytes were infected for 6 hours with *M. marinum* before a 1h treatment with amikacin and subsequent incubation for 24h and 48h. The number of bacteria was determined by CFU counts. Amikacin treatment allow to remove adherent bacteria to the surface and keep only internalized bacteria. The growth of internalized bacteria was counted after 24 and 48 hours. Each bar represents the average of three independent measurements and error bars represent standard deviations.

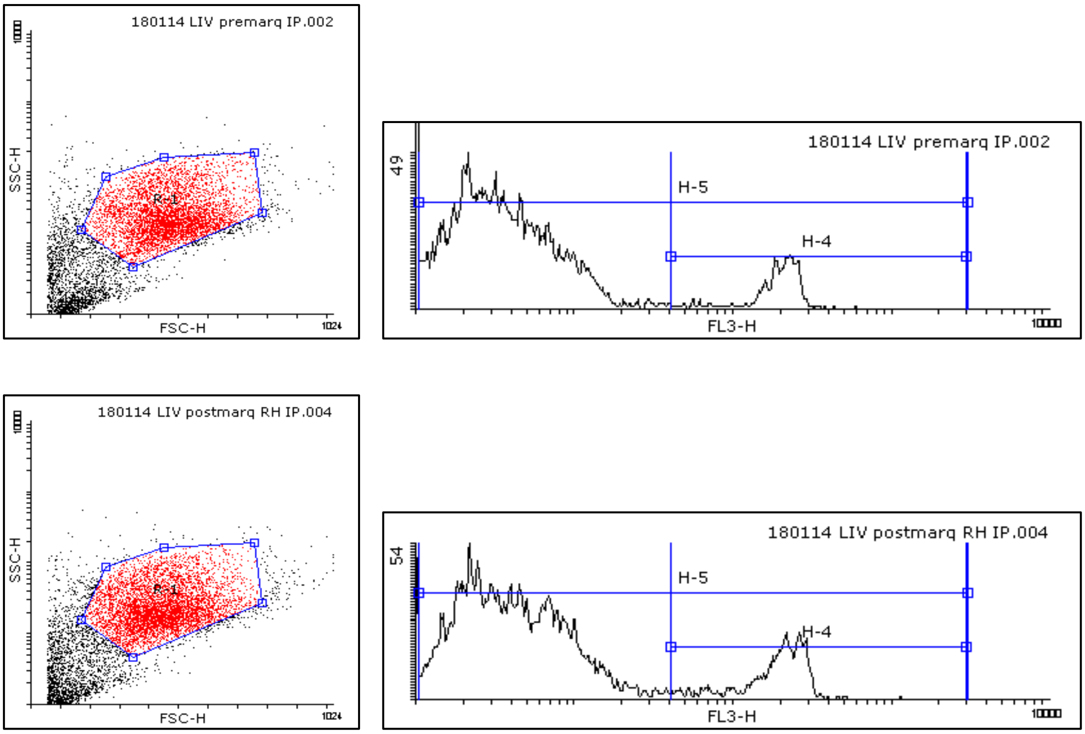

Supplementary figure 5: Staining of hemocytes with Rhodamine 123. Representative forward scatter (FCS) and side-scatter (SSC) histograms (*Left*) and fluorescence (FL3, *Right*) from PI-stained hemocytes before (*Upper histograms*) and after (*Lower histograms*) staining with rhodamine.
